# Effect of curcumin supplementation on serum expression of select cytokines and chemokines in a female rat model of nonalcoholic steatohepatitis

**DOI:** 10.1186/s13104-019-4540-5

**Published:** 2019-08-09

**Authors:** Matthew B. Pickich, Mark W. Hargrove, C. Niles Phillips, James C. Healy, Angelique N. Moore, Michael D. Roberts, Jeffrey S. Martin

**Affiliations:** 1 Department of Cell Biology and Physiology, Edward Via College of Osteopathic Medicine-Auburn Campus, Auburn, AL 36832 USA; 2 School of Kinesiology, Auburn University, Auburn, AL 36849 USA; 3 Department of Basic Medical Sciences, Debusk College of Osteopathic Medicine, Knoxville, TN 37932 USA

**Keywords:** Curcumin, Turmeric, Inflammation, NASH, NAFLD, Cytokines, Chemokines, Supplements

## Abstract

**Objective:**

We recently reported that curcumin supplementation in a metabolically (i.e., Western diet [WD]) and chemically (i.e., CCl_4_) induced female rat model of non-alcoholic steatohepatitis (NASH) was associated with lower liver pathology scores and molecular markers of inflammation. This occurred when curcumin was given during induction of disease (preventative arm; 8-week WD with or without curcumin [8WD + C vs. 8WD]) as well as when given after disease development (treatment arm; 12-week WD with or without curcumin during weeks 9–12 [12WD + C vs. 12WD]). Herein, we sought to extend our findings from that study by determining the effects of curcumin supplementation on cytokine/chemokine expression in serum collected from these same rats.

**Results:**

24 cytokines/chemokines were assayed. IL-2 (+ 80%) and IL-13 (+ 83%) were greater with curcumin supplementation in the prevention arm. IL-2 (+ 192%), IL-13 (+ 87%), IL-17A (+ 81%) and fractalkine (+ 121%) were higher while RANTES was lower (− 22%) with curcumin supplementation in the treatment arm (*p *< 0.05 for all). RANTES concentrations also correlated significantly with hepatic pathology scores of inflammation (r = 0.417, *p *= 0.008). Select serum cytokines/chemokines were affected with curcumin supplementation in this female rat model of NASH. Moreover, curcumin’s effect(s) on RANTES and its association with liver disease pathogenesis and progression may warrant further investigation.

## Introduction

The pathogenesis and progression of non-alcoholic fatty liver disease (NAFLD) and non-alcoholic steatohepatitis (NASH) is multifaceted and complex in nature, but major contributors appear to include steatosis, mitochondrial dysfunction, oxidative stress, impaired hepatocyte proliferation and inflammation [1, 2]. The main active component of turmeric (*Curcuma longa* L.)—curcumin, has been shown to have anti‐inflammatory, anti-oxidant, and anti-fibrotic properties [3, 4]. Thus, it is not surprising that the potential of curcuminoids in liver disease has been of interest [5, 6]. Indeed, preliminary studies suggest curcumin may play a beneficial role in mitigating liver disease development via anti‐inflammatory actions, as well as restoring balance to hepatic pro- and anti-oxidant systems [7–9]. Limited evidence also suggests that curcumin may have anti-steatotic [10] and anti-fibrotic effects on the liver [11, 12]. To this end, we recently evaluated the prophylactic and therapeutic potential of a turmeric extract/curcumin supplement, BCM-95^®^, in a rat model of NAFLD/NASH [13]. In that study/model, we found that curcumin supplementation was partially effective in preventing the development of liver disease and more effective in mitigating its progression. Notably, from both the preventative and treatment arms of the study, curcumin supplementation was associated with reduced molecular markers (e.g., TNF-α, SPP1 gene expression) and pathology scores of inflammation in hepatic tissue.

Given the more uniform (i.e., occurring in both the preventative and treatment arms) anti-inflammatory effects with curcumin supplementation observed in our prior report, the purpose of the present study was to expand on that work and further explore the effect of curcumin on inflammation in this metabolically and chemically induced female rat model of NASH. Accordingly, using serum collected from the rats in the aforementioned study, we assessed concentrations of a panel of cytokines/chemokines and their association with liver pathology scores. We hypothesized that curcumin supplementation would reduce inflammation associated cytokines/chemokines compared to their respective control (i.e., non-supplemented) group when administered both prophylactically (i.e., prior to/during disease development) and therapeutically (i.e., after disease development).

## Main text

### Methods

The data presented herein is an extension/continuation of previously published work [13] and the reader is referred to that publication for additional details beyond the scope of this report. Briefly, 3-month old female Wistar rats were purchased (Harlan Laboratories, Indianapolis, IN, USA) and acclimated in the animal housing facility at Auburn University for 5-month prior to initiation of the study protocol. During acclimation, rats were pair housed and provided a standard rodent chow (24% [% kcal] protein, 58% carbohydrate [2.8% fiber w/w], 18% fat; Teklad Global #2018 Diet, Harlan Laboratories) and water ad libitum in a maintained ambient temperature and constant 12 h light: 12 h dark cycle.

Following acclimation, rats were randomly assigned to one of four groups: 8-week Western diet (8WD), 8-week WD supplemented with curcumin (8WD + C), 12-week WD (12WD), and 8-week WD followed by 4-week WD supplemented with curcumin (12WD + C). All rats were (1) allowed ad libitum consumption of their respective chow, (2) provided 15% fructose drinking water (15 g/100 mL) for the duration of the study and (3) received intraperitoneal injections of carbon tetrachloride (CCl_4_ at 0.5 mL/kg body mass) at the start of weeks 1, 3, 5 and 7 during the experiment. Fructose drinking water and CCl_4_ administration in addition to a WD was utilized based on the accelerated rodent model of NALFD/NASH described by Chheda et al. [14].

The WD provided to rats was custom modified for 2% cholesterol by weight (43% fat, 34% CHO, 23% protein, TD.160279; Envigo Teklad Diets, Madison, WI, USA). The same WD was supplemented with curcumin (BCM-95^®^; Dolcas Biotech, LLC, Landing, NJ, USA) at 0.2% of weight (TD.160280; Envigo Tekland Diets) for respective groups/diets. Curcumin concentration in the respective chow (i.e., 0.2%) was chosen targeting a dose of ~ 100 mg/kg/day of body mass, similar to therapeutic doses in human consumption, and based on our historical observations of daily chow consumption rates in similarly aged female Wistar rats. The curcumin supplement was reported to contain 70% curcumin, 17% demethoxycurcumin, 3.5% bis-demethoxycurcumin and 7.5% turmeric essential oils. Body weight, chow and fructose drinking water consumption were measured on a weekly basis.

#### Necropsies and tissue preparation in rats from both feeding experiments

The night before necropsies, rats had their respective chow removed from their cage, but were provided tap water ad libitum in order to facilitate overnight fasting conditions. The following morning, rats were transported to the lab and allowed to acclimate to laboratory conditions for 2–3-h. Thereafter, rats were euthanized (CO_2_ gas in a 2 L induction chamber; VetEquip, Pleasanton, CA, USA), a final body mass was recorded, and blood was collected from the heart using a 22-gauge syringe. Blood was placed in a 6-mL serum separator tube and allowed to clot, centrifuged at 3500*g* for 10-min, and resultant serum was aliquoted into 1.7-mL microcentrifuge tubes for storage at − 80 °C until analysis. Liver tissues were dissected out and masses were recorded using a calibrated scale with a sensitivity of 0.0001 g (Mettler-Toledo; Columbus, OH). A segmented piece of the liver tissue (~ 100 mg) was placed in a conical tube containing 5 mL of 10% formalin for histology.

#### Liver pathology

Formalin fixed liver specimens were transferred to Veterinary Diagnostics Pathology, LLC (Fort Valley, VA, USA) for analysis. To examine liver morphology, formalin-fixed, paraffin-embedded livers were sectioned and stained with hematoxylin and eosin (H&E). Liver specimens were graded and scored by an independent pathologist for changes in fat accumulation, ballooning, inflammation and fibrosis. These scores are presented in our previously published work [13].

#### Serum analyses

Serum concentrations of cytokines/chemokines were determined using a commercially available 96-well multiplex assay kit according to manufacturer’s instructions (RECYMAG65K27PMX, EMD Millipore Corporation, Billerica, MA, USA). Of note, 2 of the assay targets in the multiplex assay, IFNg and GRO/KC/CINC-1, are not reported herein due to poor signal and/or being below the sensitivity of the assay.

#### Statistical analyses

Separate analyses were performed for each arm of the study; 8WD vs. 8WD + C to determine the effects of curcumin supplementation from a preventative standpoint and 12WD vs. 12WD + C to determine the effects of curcumin from a treatment (i.e., already established disease) perspective. Levene’s Test for equality of variances was performed for all data and Student’s independent or Welch–Satterthwaite t-tests were employed as appropriate. Pearson’s correlations between significantly altered cytokines/chemokines and previously reported pathology scores were also performed. Data are presented as mean ± standard deviation (n = 7–12 observations are represented per group). All statistical analyses were completed using SPSS (IBM SPSS Statistics for Windows, Version 24.0. Armonk, NY, USA) with *p* < 0.05 used to determine statistical significance.

### Results

#### Animal characteristics

One rat in the 8WD + C died unexpectedly (unknown reason) and three additional rats (2 from 12WD and 1 from 12WD + C groups) were euthanized early for humane reasons (significant weight loss/tumor). Animal characteristics for all remaining animals by group have been reported previously [13]. In brief, body mass (initial and final), food intake, caloric intake and liver mass were not different from respective controls for the prevention (i.e., WD8 vs. WD8 + C) and treatment (i.e., WD12 vs. WD12 + C) arms of the study. Food intakes revealed average curcumin (i.e., BCM-95) consumption at 68 ± 27 mg/kg/day and 75 ± 10 mg/kg/day for rats in the WD8 + C and SD12 + C groups, respectively.

#### Effect of curcumin supplementation on serum cytokines and chemokines

**Fig. 1:**
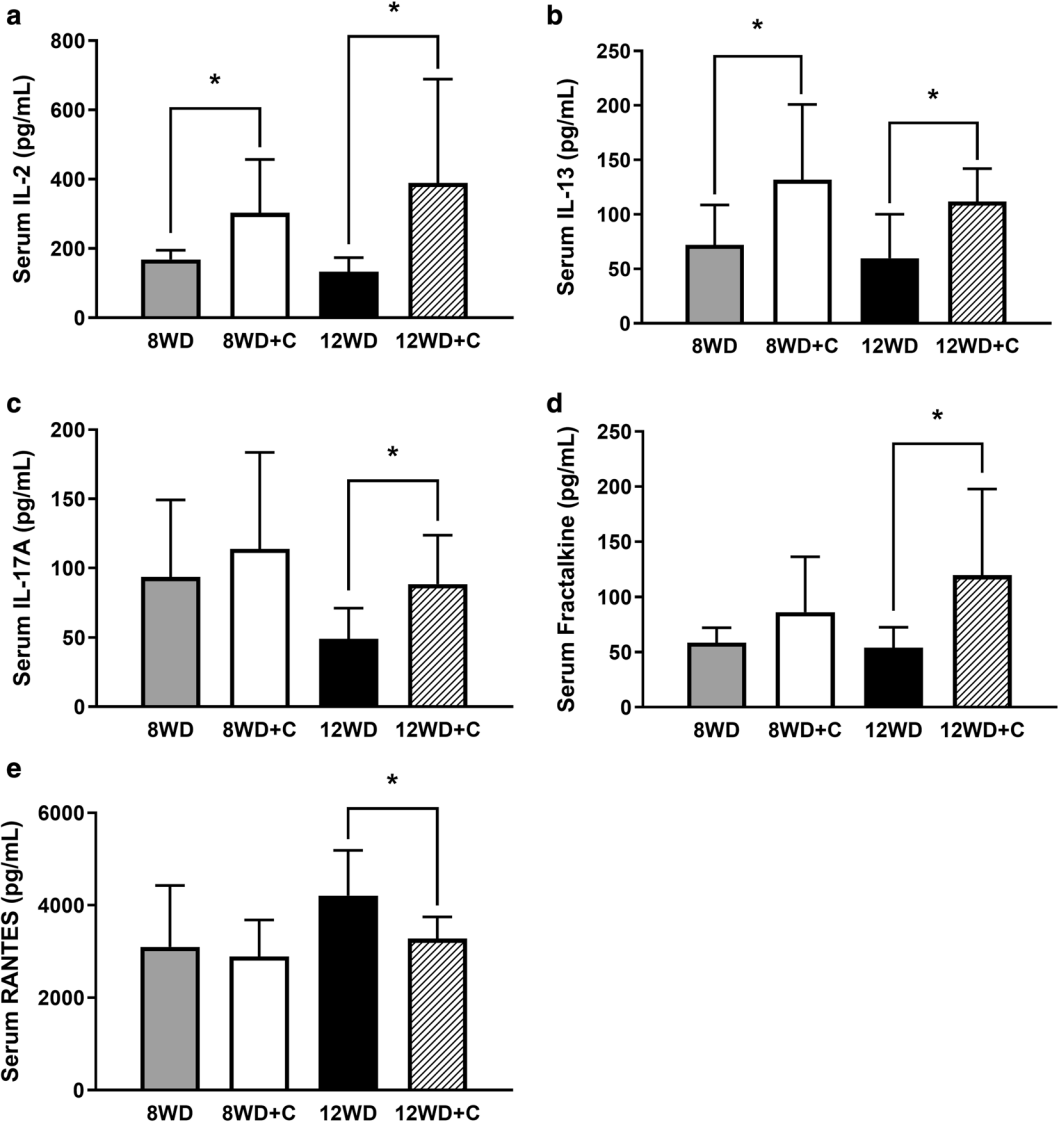
Serum. **a** Interleukin (IL)-2, **b** IL-13, **c** IL-17A, **d** fractalkine, and **e** RANTES concentrations at sacrifice in the prevention (8WD, 8-week Western diet vs. 8WD + C, 8-week WD + curcumin) and treatment (12WD, 12-week Western diet vs. 12WD + C, 12-week WD + curcumin during weeks 9–12) arms of the study. Values are mean concentration ± SD. *Significantly different between groups (*p *< 0.05)

In the prevention arm of the study, only two of the assay targets, interleukin (IL)-2 and IL-13, were found to be differentially expressed in serum with curcumin supplementation (8WD + C) compared to the control/non-supplemented group (8WD). Significantly higher concentrations of both IL-2 (+ 80%, *p *= 0.020; Fig. 1a) and IL-13 (+ 83%, *p *= 0.038; Fig. 1b) were found in the 8WD + C group compared to the 8WD group.

In the treatment arm of the study, five targets were expressed at significantly different levels between the 12WD and 12WD + C groups. Serum expression of IL-2 (+ 192%, *p *= 0.025), IL-13 (+ 87%, *p *= 0.017), IL-17A (+ 81%, *p *= 0.011) and fractalkine (+ 121%, *p *= 0.028) were all significantly higher in the 12WD + C group compared to the 12WD group (Fig. 1a–d). In contrast, serum RANTES concentrations were found to be significantly lower in the 12WD + C group compared to the 12WD group (− 22%, *p *= 0.025; Fig. 1e).

All additional cytokine/chemokine targets did not differ in serum concentration between groups and are reported in Table 1 (*p *> 0.05 for all).

**Table 1:** Serum concentrations of select cytokines/chemokines at sacrifice

Cytokine/chemokine	8WD (n = 9–12)	8WD + C (n = 9–11)	12WD (n = 7–9)	12WD + C (n = 8–11)
EGF (pg/mL)	3.52 ± 3.04	5.63 ± 3.50	7.79 ± 13.71	6.33 ± 4.86
Eotaxin (pg/mL)	43.2 ± 17.6	56.7 ± 24.6	41.6 ± 23.0	59.2 ± 36.2
G-CSF (pg/mL)	98 ± 35	153 ± 87	100 ± 52	100 ± 43
IL-1α (pg/mL)	289 ± 159	321 ± 257	275 ± 355	288 ± 203
IL-1β (pg/mL)	133 ± 83	147 ± 94	107 ± 61	136 ± 84
IL-4 (pg/mL)	132 ± 88	153 ± 109	92 ± 80	121 ± 66
IL-5 (pg/mL)	363 ± 114	347 ± 101	324 ± 195	527 ± 308
IL-6 (ng/mL)	1.46 ± 1.37	1.82 ± 1.46	0.64 ± 0.50	1.12 ± 0.88
IL-10 (pg/mL)	109 ± 63	118 ± 91	85 ± 39	146 ± 125
IL-12p70 (pg/mL)	808 ± 375	882 ± 466	599 ± 99	681 ± 145
IL-18 (pg/mL)	342 ± 217	470 ± 293	340 ± 272	425 ± 255
IP-10 (pg/mL)	296 ± 78	274 ± 56	369 ± 72	375 ± 80
Leptin (ng/mL)	38.8 ± 21.6	51.4 ± 28.6	54.2 ± 27.5	50.0 ± 24.7
LIX (ng/mL)	3.54 ± 0.89	3.18 ± 1.25	3.82 ± 0.60	4.00 ± 0.55
MCP-1 (ng/mL)	1.75 ± 0.34	1.70 ± 0.57	1.48 ± 0.31	1.66 ± 0.39
MIP-1α (pg/mL)	35.5 ± 11.3	38.5 ± 13.9	32.5 ± 6.1	39.9 ± 13.4
MIP-2 (pg/mL)	112 ± 39	142 ± 38	101 ± 48	163 ± 139
TNFα (pg/mL)	93 ± 50	103 ± 61	69 ± 27	94 ± 42
VEGF (pg/mL)	63.1 ± 33.1	75.8 ± 34.6	61.4 ± 23.9	99.6 ± 61.0

Values are mean ± SD

*EGF* epidermal growth factor, *G-CSF* granulocyte-colony stimulating factor, *IL* interleukin, *LIX* lipopolysaccharide-inducible CXC chemokine, *MCP-1* monocyte chemoattractant protein-1, *MIP* macrophage inflammatory protein, *TNFα* tumor necrosis factor alpha, *VEGF* vascular endothelial growth factor

## Discussion

The primary finding of this study is that of the 24 cytokines/chemokines explored, serum concentrations were significantly affected, in one or both arms of the study (prevention and/or treatment), for five of the targets (~ 21%). Four of those targets, IL-2, IL-13, IL-17A and fractalkine, were in higher concentration with curcumin supplementation while only one, RANTES, was in lower concentration with curcumin supplementation.

We previously reported that curcumin supplementation was associated with lower histological liver inflammation scores, AST levels, and inflammation (i.e., TNF-α, SPP1 mRNA) and fibrosis associated gene expression (i.e., Col1a1 mRNA) in both the prevention and treatment arms of the study [13]. Herein, both IL-2 and IL-13 were found to be significantly higher on average in curcumin supplemented rats (8WD + C and 12WD + C) compared to their respective control group (8WD and 12WD). Given that IL-13 down-regulates macrophage activity and inhibits production of pro-inflammatory cytokines and chemokines [15], this finding was not surprising in the context of the previously reported findings [13]. However, it was notable to see higher levels of IL-2 in curcumin supplemented rats as curcumin has been shown to suppress IL-2 production [16] and inactivate NF-κB [17]. While speculative, perhaps decreased downstream activity mediated by curcumin in the effector pathway(s) reduces negative feedback on cytokine production [18] and/or there is some benefit to elevated IL-2 concentrations secondary to the metabolic and chemical (i.e., CCl_4_) liver tissue injury given that some cytokines/chemokines also mediate liver tissue regeneration after injury [19]. Nevertheless, the exact role of curcumin in augmenting these cytokines/chemokines and their role, or lack thereof, in phenotypic outcomes in this rat model of NAFLD/NASH should be further elucidated.

**Fig. 2:**
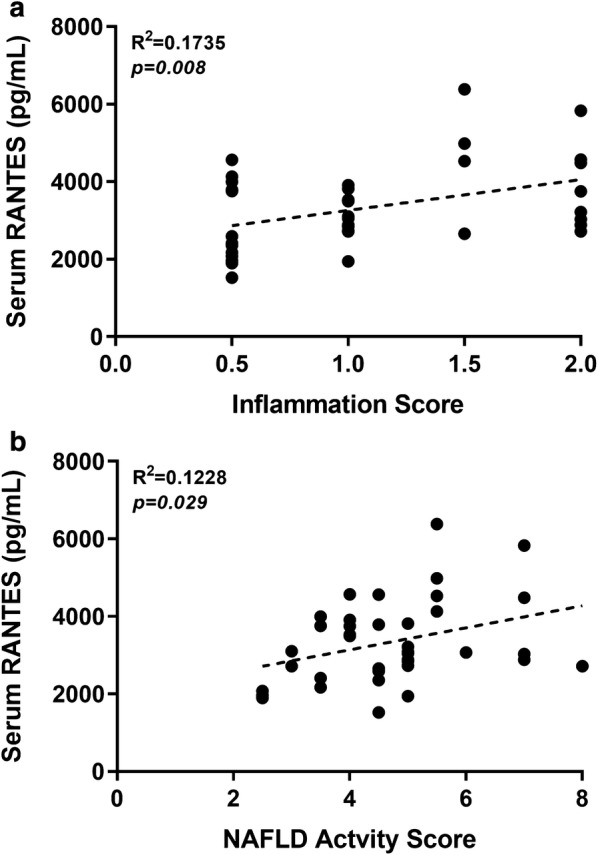
Pearson’s correlations between serum concentrations of RANTES and pathology scores for **a** inflammation and **b** non-alcoholic fatty liver disease (NAFLD) activity scores. Each data point represents an individual rat and data for both arms of the study are shown

Specific to the treatment arm of this study, serum fractalkine and IL-17A were also found to be expressed in higher concentrations with curcumin supplementation (12WD + C vs. 12WD). Fractalkine may play an anti-inflammatory and anti-fibrotic role in the liver [20] though IL-17A is a pro-inflammatory cytokine [21, 22]. Notably, although associated with NAFLD/NASH pathogenesis [22], IL-17A appears to mediate its effects through MAPK, NF-κB, and AP-1 which have all shown to be inhibited by curcumin [23]. Thus, while again speculative, perhaps a reduction in feedback and/or stage of the disease state (i.e., less fibrotic tissue) perpetuates greater IL-17A production. Finally, in the treatment arm of the study, we observed significantly lower serum levels of RANTES in the curcumin supplemented animals. RANTES (CCL5) is a chemokine for which increased expression, from hepatocytes, has been associated with pathogenesis and progression of NAFLD/NASH [24]. Moreover, increased expression of RANTES has been observed in models of both toxic (i.e., CCl_4_) [25] and diet [24] induced liver injury. Herein, RANTES was the only chemokine/cytokine of those that were significantly altered by curcumin supplementation to (1) be in lower concentration and (2) correlate with our previously published pathology findings in these rats [13]. Indeed, across both arms of the study, serum RANTES was positively correlated with both inflammation (r = 0.417, *p *= 0.008; Fig. 2a) and NAFLD activity (r = 0.350, *p *= 0.029; Fig. 2b) scores. While these data are in rodents, this finding in particular is interesting with respect to not only the actions of curcumin but also the possibility that serum RANTES levels could be a viable biomarker to monitor the progression of NAFLD/NASH.

Of note, the proximity of CCl_4_ administration to necropsies was not uniform across arms of the study and may have impacted the potential actions/effects of curcumin. Moreover, this may explain the greater number of cytokines/chemokines affected as well as the more consistent phenotype in the treatment arm of the study. Indeed, along with the lower histological inflammation scores, AST levels and inflammatory/fibrotic gene expression observed in both arms of the study with curcumin supplementation, lower NAFLD activity scores, ALP levels and hepatic FGF-21 levels were also observed in the treatment arm [13]. Thus, comparisons between the two arms of the study are difficult (and were not made) given the marked toxicity of this compound [26]. Nevertheless, this study continues to elucidate the complex phenotype of NAFLD/NASH, particularly in this metabolic and chemically induced female model, while also providing serum biomarkers that are affected with curcumin supplementation.

## Limitations

Limitations to the present report include the fact that over 20 cytokines/chemokines were evaluated, but adjustment for multiple comparisons was not performed given the exploratory nature of the work. In addition, we utilized only female rats as they were reported to be more susceptible to disease development than males in this model [14]. Sex differences in the pathogenesis of liver disease have also been demonstrated in an alternate nutritional model of NASH [27]. Thus, it unknown how the results herein would compare to a population of male rats and the homogeneity of effects from curcumin supplementation across sexes in this model should be further explored. Finally, the model we utilized was for NASH, which includes significant inflammation in its pathogenesis, but is not a specific, focused inflammatory model. Future studies comparing the outcomes herein in CCl_4_ absent and/or more inflammation-focused models would be of interest.

## Data Availability

Datasets used and analyzed for the current study are available from the corresponding author(s) upon reasonable request and with permission from funders (Dolcas Biotech, Arjuna Naturals).

## Abbreviations

NAFLD: non-alcoholic fatty liver disease
NASH: non-alcoholic steatohepatitis
IL: interleukin
CCl_4_: carbon tetrachloride
WD: Western diet
8WD: 8-week Western diet
8WD + C: 8-week Western diet supplemented with curcumin
12WD: 12-week Western diet
12WD + C: 12-week Western diet supplemented with curcumin in weeks 9–12
